# Supplementary material for: The Conserved Cysteine-Rich Secretory Protein MaCFEM85 Interacts with MsWAK16 to Activate Plant Defenses
Source: Int J Mol Sci. 2023 Feb 17;24(4):4037. doi: 10.3390/ijms24044037 (PMC9967070; doi:10.3390/ijms24044037)
Supplement: Supplementary file 1 [file ijms-24-04037-s001.zip › Supplementary Table S1.pdf]

Strains and plasmids used in this study

**strains/plasmid**		
Escherichia coli		
DH5a	High efficiency transfo	Lab collection
BL21(DE3)	Specifically constructe	Lab collection
Agrobacterium		
GV3101	Wild-type, Rif$^{R, 1}$	Lab collection
Yeast strain		
Gold		Clontech Co., Ltd.
Plasmids		
PYBA1132-eGFP	Transient expression vector for eGFP in protoplast, Ka^{R,3}	
eGFP-MaCFEM85		This study
pCAMBIA1300-mChe	Transient expression vector for mCherry in protoplast, Ka^{R,3}	
mCherry-MaCFEM85		This study
pGADT7	Expression vector in Yeast for Y2H assay, Amp^{R}	
pGADT7-MsWAK16-ED		This study
pGBKT7	Expression vector in Yeast for Y2H assay, Ka^{R}	
pGBKT7-MaCFEM85^{NSP}		This study
pGBKT7-MaCFEM85-C		This study
pGBKT7-MaCFEM85-CFEM		This study
pGBKT7-ΔCFEM85$_{26}$		This study
pGBKT7-ΔCFEM85$_{30}$		This study
pGBKT7-ΔCFEM85$_{43}$		This study
pGBKT7-ΔCFEM85$_{52}$		This study
pUC-SPYCE	Expression vector in Nicotiana benthamiana for BiFC assay, Amp^{R}	
pUC-SPYCE -MsWAK16		This study
pUC-SPYNE	Expression vector in Nicotiana benthamiana for BiFC assay, Amp^{R}	
pUC-SPYNE-MaCFEM85		This study
fungus strain		
Botrytis cinerea		Lab collection
Metarhizium anisopliae		Lab collection
