# Supplementary material for: The Conserved Cysteine-Rich Secretory Protein MaCFEM85 Interacts with MsWAK16 to Activate Plant Defenses
Source: Int J Mol Sci. 2023 Feb 17;24(4):4037. doi: 10.3390/ijms24044037 (PMC9967070; doi:10.3390/ijms24044037)
Supplement: Supplementary file 1 [file ijms-24-04037-s001.zip › Supplementary Table S2.pdf]

The designed primers used in this study

Purpose of use	Primer name	Primer sequence
Conservative analysis		
Subcellular localization	MaCFEM85-PYBA1132-F	caATTGGAGCTCCACCGCATGCGATCCTCATTCGTC
	MaCFEM85-PYBA1132-R	GGTACCGGGCCCCCCCTC GAGAGCGGCAACGATGGCC
	MaCFEM85-mcherry-F	ggagaga acacgggggacATGCGATCCTCATTC
	MaCFEM85-mcherry-R	CAGGATCCCGGGCCCGCGGAGAGCGGCAACGATGGCC
Y2H	MsWAK16-ED-pGADT7-F	ttacgctcatatggccatgACAACTCAGCAACTATCTCT
	MsWAK16-ED-pGADT7-R	GCAGCTCGAGCTCGATGG CATTATGATCTCTTTCCTTG
	MaCFEM85-NSP-pGBKT7-F	aggaggacctgcatatgCAGCTCGGCGAAATCCCC
	MaCFEM85-NSP-pGBKT7-R	GACCCGTTTAGAGGCCCTTAGAGAGCGGCAACGATGG
	MaCFEM85-CFEM-pGBKT7-F	aggaggacctgcatatg CTCGGCGAAATCCCCTCATG
	MaCFEM85-CFEM-pGBKT7-R	GACCCGTTTAGAGGCCC GGCGGCACACAGGCCGGTAGC
	MaCFEM85-C-pGBKT7-F	aggaggacctgcatatg AGCGGCGTCGACACTCCCAA
	MaCFEM85-C-pGBKT7-R	GACCCGTTTAGAGGCCC GAGAGCGGCAACGATGGC
	MaCFEM85-ΔCFEM85$_{26}$-pGBKT7-F	aggaggacctgcatatgCTCGGCGAAATCCCCTCAGCAGC
	MaCFEM85-ΔCFEM85$_{26}$-pGBKT7-R	GACCCGTTTAGAGGCCC GGCGGCACACAGGCCGGTAGC
	MaCFEM85-ΔCFEM85$_{30}$-pGBKT7-F	aggaggacctgcatatgCAGCTCGGCGAAATCCCC
	MaCFEM85-ΔCFEM85$_{30}$-pGBKT7-R	GACCCGTTTAGAGGCCC GGCGGCACACAGGCCGGTAGC
	MaCFEM85-ΔCFEM85$_{43}$-pGBKT7-F	aggaggacctgcatatgCAGCTCGGCGAAATCCCC
	MaCFEM85-ΔCFEM85$_{43}$-pGBKT7-R	GACCCGTTTAGAGGCCC GGCGGCACACAGGCCGGTAGC
	MaCFEM85-ΔCFEM85$_{52}$-pGBKT7-F	aggaggacctgcatatgCAGCTCGGCGAAATCCCC
	MaCFEM85-ΔCFEM85$_{52}$-pGBKT7-R	GACCCGTTTAGAGGCCC GGCGGCACACAGGCCGGTAGC
	MaCFEM85-ΔCFEM85$_{8\text{ all}}$-pGBKT7-F	aggaggacctgcatatgCTCGGCGAAATCCCCTCAGCAGC
	MaCFEM85-ΔCFEM85$_{8\text{ all}}$-pGBKT7-R	GACCCGTTTAGAGGCCC GGCGGCTGCCAGGCCGGT
BiFC	MaCFEM-NE-F	gagttaaccgggctcaggccATGCGATCCTCATTCGTC
	MaCFEM-NE-R	GTACCCTCGAGGTCGACAGTGAGAGCGGCAACGATGGCC
	MsWAK16-CE-F	GAGTTAACCGGGCTCAGGCCATGCACATGGCTGTGGCCTC
	MsWAK16-CE-R	GTACCCTCGAGGTCGACAGTTCTTCCACCAACAAATGAC
pull down	MaCFEM-NSP-21b-F	CAGCAAATGGGTCGGGATCAGCTCGGCGAAATCCCC
	MaCFEM-NSP-21b-R	TGGTGCTCGAGTGCGGCCGCGAGAGCGGCAACGATGGCC
	MsWAK16-ED-6P2-F	GTTCTGTTCCAGGGGCCC ACAACTCAGCAACTATCTCT
	MsWAK16-ED-6P2-R	CAGAGGTTTTCACCGTCATC CATTATGATCTCTTTCCTTG
qRT-PCR primer	q WAK16-F	CAGGATGTTTGGCTTTGTGC
	q WAK16-R	CTTGAGGAGGTCCGTGGATT
	q-CFEM85-F	CAGCTCGGCGAAATCCCCTCAT
	q-CFEM85-R	TGGGAGTGTCGACGCCGCTG
	Ma-Try-F	TTGCAATGCATGTTTGATGTC
	Ma-Try-R	CAAAGAGTGGTATCGAGTTAC

Ms-Actin-F	ACTGGAATGGTTAAGGCTGG
Ms-Actin-R	CCTCTTAGACTGTGCCTCATC
q-GFP-F	ACCATCTTCTTCAAGGACGAC
q-GFP-R	CGTTGTGGCTGTTGTAGTTGT
N.b-CM-F	TCAGCCTTGTGGAGAGAGTTC
N.b-CM-R	GGAAGAATGGGTGCTCATCA
N.b-ADT-F	TAGCAAGGTTGTCTCGCAGC
N.b-ADT-R	CGCAAGGTATGGCTTCACAT
N.b-PAT-F	CACTTTGTGCTGCCTTTAGG
N.b-PAT-R	CATCCGCAATCACTATGGTC
N.b-CHS-F	TGGCACCTTCTCTTGATGC
N.b-CHS-R	TGAGTTGGTAGTCACACCCG
N.b-4CL-F	AACTTGGTCAGGGTTATGGG
N.b-4CL-R	GATTTGGTCACCTCGTATGC
N.b-WAK70-F	AATCAGCGTTTGAGAGGGA
N.b-WAK70-R	AGGCTTTGCTTCCACAATCT
N.b MYC2-F	TTCTGCTGCTGCTATTACTGC
N.b MYC2-R	CAATGAGGGTTTGGAGACG
N.b-PDF1.2-F	GGCTACAGAGATGGGACCAA
N.b-PDF1.2-R	AAACAGACGGTGGCACAGTT
N.b-Actin-F	CGAGGGTTATGCTTTGCCTC
N.b-Actin-R	AGCAAGCTCCTCCTTCATGT
N.b-PR1a-F	GATGCCCATAACACAGCTCG
N.b-PR1a-R	AGCAAGCTCCTCCTTCATGT
N.b-PAL-F	ATTGCTGGTTTGCTCACTGG
N.b-PAL-F	TCCTTAGGCTGCAACTCGAA
N.b-NPR1-F	GATACACGGTGCTGCATGTT
N.b-NPR1-R	AAGCCTAGTGAGCCTCTTGG
N.b-COI1-F	AACTGGTCGGGATCTCTTGG
N.b-COI1-R	TAGGCAAGTATATGGGCGGG
