# Supplementary figures and images for: The Conserved Cysteine-Rich Secretory Protein MaCFEM85 Interacts with MsWAK16 to Activate Plant Defenses
Source: Int J Mol Sci. 2023 Feb 17;24(4):4037. doi: 10.3390/ijms24044037 (PMC9967070; doi:10.3390/ijms24044037)

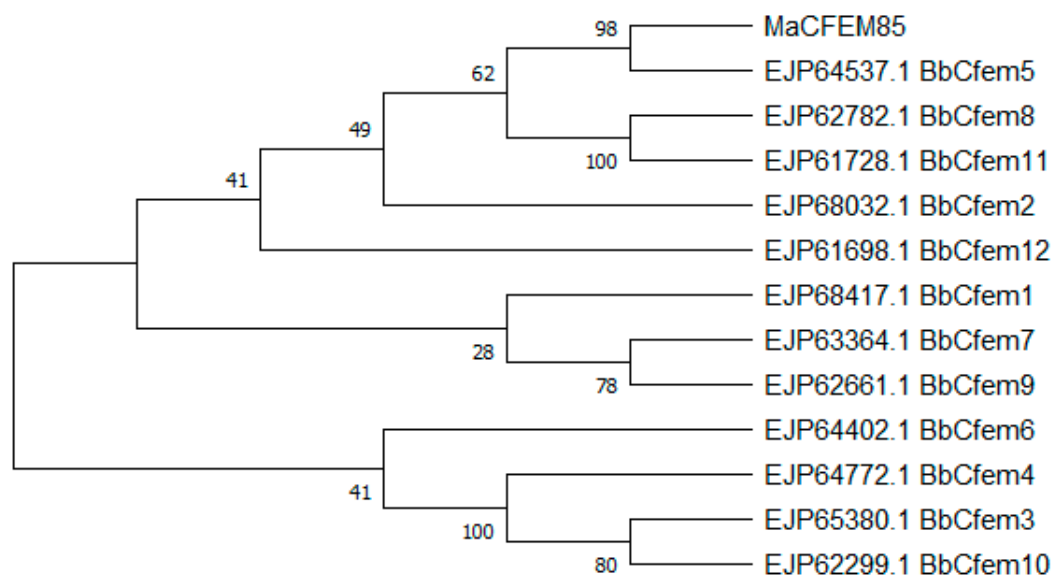

**Figure S1.** Phylogenetic analysis of MaCFEM85 with *Beauveria bassiana* 12 CFEM proteins.

Supplement: Supplementary file 1 [file ijms-24-04037-s001.zip › Supplementary Figure S1.pdf]
